# Family perceptions of intellectual disability: Understanding and support in Dar es Salaam

**DOI:** 10.4102/ajod.v1i1.32

**Published:** 2012-10-04

**Authors:** Heather M. Aldersey

**Affiliations:** 1 University of Kansas Beach Center on Disability, University of Kansas, Lawrence, USA

## Abstract

When attempting to understand the construct of intellectual disability in different contexts, speaking to family members in addition to the individual with the disability may provide new insight about understandings of and responses to intellectual disability in society and may help to identify the forms of support that are available or needed to ensure the quality of life of people with disabilities. This article outlines and discusses interviews that were conducted in Dar es Salaam, Tanzania, with family members of children and adults with intellectual disabilities. These interviews explore how families came to understand that their child had an intellectual disability; the availability of family support; and family hopes and dreams for the future, and were a part of a wider exploratory study that gathered insight from individuals with disabilities, families, and other providers of support to explore understandings and perceptions of disability in Dar es Salaam. Understanding family experiences will help researchers, policy makers, non-governmental organisations, and others to identify family strengths and family support needs which can ultimately improve family quality of life and the quality of life of the member with a disability.

## Introduction

In most nations worldwide, the family is the first and most enduring unit of society and is usually the primary source of influence behind the formation of personality and the growth of an individual (Macionis 2011). For the lives of people with intellectual disabilities, family members often continue to provide invaluable support throughout their lifespan (Chou, Lin, Chang, & Schalock 2007; Hill & Rose 2009; McConkey 2005). When attempting to understand the construct of intellectual disability in various different contexts, speaking to family members, in addition to the individual with a disability, may provide different insight about understandings of and responses to intellectual disability in society. It may help to identify the supports that are available or needed to ensure the quality of life of the family member with a disability.

### Statement of the problem

In this study, my research questions were (a) how do families conceptualise and experience disability?; (b) what are families greatest support needs?; and (c) what are the unique strengths of each family? I aimed to expand upon Mbwilo *et al*.’s (2010) important exploration of family and intellectual disability in Dar es Salaam by further probing family understanding of intellectual disability and identifying areas of family support needs while also providing a discussion of family strengths. After identification of family strengths, one can attempt to build upon those strengths to improve overall family quality of life.

In this article, I discuss the results of interviews conducted with family members of children and adults with intellectual disabilities in Dar es Salaam, Tanzania. These interviews explore how families came to understand that their child had an intellectual disability; the availability of family support; and family needs, hopes, and dreams for the future. The data gathered through the interviews were a part of a wider exploratory study that gathered insight from individuals with disabilities (including intellectual, physical, and sensory disabilities), families, and other providers of support to explore understandings and perceptions of disability in Dar es Salaam. Understanding family experiences will help researchers, policy makers, non-governmental organisations, and others to identify family strengths and support needs which can ultimately improve family quality of life and the quality of life of the member with a disability.

Family is an important aspect in the lives of people with disabilities because the perceptions of disability, as well as the roles assigned to individuals with a disability, are greatly informed by the family structure and the person’s place within the family (Rao 2006). Family comes to play an even more important role in countries without extensive professional services. Turnbull and Turnbull (2001) assert that when individuals with an intellectual disability are unable to make the decisions in their lives without assistance, that assistance should come from trusted allies – those people who are deeply committed and have genuine emotional relationships with the person. For this study, I defined a family member to be any person who is affiliated with the person with a disability by consanguinity, affinity, or coresidence.

Disability is a cultural creation: disability status depends less on the nature or degree of a person’s impairment and more on societal standards for normative bodies, minds, behaviours, and roles (Armstrong & Fitzgerald 1996; Ingstad & Whyte 1995). Although the definition of intellectual disability in a specific Tanzanian cultural context requires further research and investigation, for the purposes of this exploratory study, I used a commonly-accepted definition of intellectual disability: someone whose intellectual function and adaptive behaviour (everyday social and practical skills) differ significantly from what is normative in his or her society (AAIDD 2011).

Finally, this study attempted to identify family strengths as well as needs. Although researchers have begun to study family needs in Tanzania (Mbwilo, Smide & Aarts 2010), there has not yet been an extensive exploration of family strengths. Even though there are limited data on family strengths in a Tanzania-specific context, researchers such as those who developed the *Family Strengths Model* (Stinnett & DeFrain 1985) have identified family strengths which have proven to be more or less universally applicable worldwide. In this article, I define family strengths as ‘the set of relationships and processes that support and protect families and family members, especially during times of adversity and change [and that] help to maintain family cohesion while also supporting the development and well-being of individual family members’ (Moore, Chalk, Scarpa & Vandivere 2002, p. 1). Internationally-relevant examples of family strengths include commitment, appreciation and affection, positive communication, time together, spiritual wellbeing, and the ability to cope with stress and crisis (DeFrain 1999).

Support can often be provided to families in order to build or enhance their strengths. In the context of this study, support is ‘resources and strategies that aim to promote the development, education, interests, and personal well-being of a person’ (Luckasson 1992:151). Family support encompasses the individualised determination of each family's needs, strengths, and preferences as the basis for accessing resources (i.e., emotional, informational, financial, and instrumental) to enhance family quality of life.

### Family perceptions of and experiences with caregiving

Although the key focus of this study was to identify family strengths and support in Tanzania, the literature on family perceptions of and experiences with caregiving of family members with intellectual disabilities globally also adds useful context to a discussion of family responses to disability, family strengths, and family support needs. It should be noted that most of the literature on caregiving comes from minority (Western) world contexts and may not be completely relevant to a Tanzanian situation. In spite of this fact, this literature can still provide background into the caregiving experience in general to which one may begin to determine the ways in which the Tanzanian experience is similar or different.

Until recently, research on caregiving of children and adults with intellectual disabilities tended to highlight the ‘burden’ of caregiving for parents (Hassall, Rose & McDonald 2005; Kenny & McGilloway 2007; Salovita, Italinna & Leinonen 2003). Researchers have identified family conflict, exhaustion, guilt, financial strain, and constricted social lives as negative outcomes of caregiving (Pearlin, Mullin, Semple & Skaff 1990; Gona, Mung’ala-Odera, Newton & Hartley 2010) and have identified support, respite care, and future planning as key areas of family concern (Dillenburger & McKerr 2010). Numerous studies have chronicled the stress and burnout associated with caring for a person with intellectual disability, with carers often presenting lower morale and greater levels of depression than the general population (Blacher, Neece & Paczkowski 2005; Blacher & McIntyre 2006). In spite of the increased levels of stress shown in parents of children with these disabilities, many parents and families of such children are well-adapted and appear resilient in the face of challenges (Gerstein *et al*. 2009)

In recent years, researchers have shown that, in addition to the challenges, there can be many positive and rewarding aspects of providing care for family members with intellectual disabilities, such as an increased sense of psychological wellbeing (Hong & Seltzer 1995). Moreover, when trying to understand the circumstances or meaning behind having a child with a disability, researchers report that many parents take comfort in their spirituality, which helps them view their child as a blessing or a test of their faith, rather than as a burden (Blacher, Neece & Paczkowski 2005). Many discussions of caring for people with intellectual disabilities combine ideas of both the ‘burden’ or stress entailed as well as its positive aspects. For example, although Kelly and McGilloway (2007) found evidence of caregiver strain, they also found that most participants were satisfied with their lives, used positive coping strategies, and had realistic expectations for their children and their future. In addition to studying the effects on families (both positive and negative) of caring for a disabled family member, research has also explored certain family traits that may affect the outcome of a their experiences. For example, in a study of hope as a psychological resilience factor in parents of children with disabilities, Lloyd and Hastings (2009) found that hope predicted increased positive wellbeing of families and decreased their psychological distress. Having outlined the nature of this study and discussed caregiving in general, I will now turn to a discussion of disability in Tanzania.

#### Dar es Salaam, Tanzania

A 2009 Tanzania National Bureau of Statistics survey showed that, in 2008, approximately 2.4 million people (or 8% of the population) experienced some type of limitation of activity and that rates of school enrolment among children with disabilities were much lower than the national school enrolment for all children. In addition to national statistics on the situation of people with disabilities, non-governmental organisations have also begun to gather data on the experiences of people with disabilities in Tanzania. For example, a 2010 assessment of the proportion of persons with disabilities in the workplace in Dar es Salaam, conducted by Comprehensive Community Based Rehabilitation in Tanzania (CCBRT), Radar Development, and Disability Aid Abroad found that of the 126 companies surveyed (with a total of 25 446 employees), only 186 or 0.7% of employees were persons with disabilities (Kweka 2010). This percentage clearly falls far short of adequately representing the percentage of the Tanzanian population living with disabilities.

In spite of the somewhat depressing statistics about the inclusion of people with disabilities in such community areas as schools and the workplace, Tanzania has an impressive history of including people with disabilities in national policy documents. Indeed, the government of Tanzania has affirmed the rights of people with disabilities since it passed the Disabled Persons Employment Act in 1982. Disability groups under the umbrella of Shivyawata, the Disabled Persons’ Organizations Federation, have often served as a positive catalyst and key advocates and advisors in the Tanzanian disability policy process. Although the Tanzanian government stands out as an example in Africa for public commitment to people with disabilities via policy, much of the Tanzanian policy concerning rights and services for people with disabilities, such as the 2004 National Policy on Disability, have been problematic because it has typically lacked specific accountability measures that would ensure that national promises are actually carried out in practice (Aldersey & Turnbull 2011).

Recently, Tanzania has taken a great step in the direction of increased accountability measures with the passing of the Disability Act of 2010 (United Republic of Tanzania 2010). As it relates to family, this act does not explicitly highlight the importance of providing support for the family. Explicit references to family made in this Act are in Article 16 and 17, which affirm the collective obligation of relatives to provide social support to their family members with disabilities and enables a person with a disability to utilise legal mechanisms to require monthly financial payments from relatives to the individual with a disability if they do not provide support voluntarily. Although provisions to support the entire family are absent in this Act, the Act does make provisions for healthcare, social support, accessibility, rehabilitation, education and vocational training, communication, employment or work protection, and promotion of basic rights for individuals with disabilities. These provisions are supplemented with such accountability measures that a 3% quota of persons with disabilities be employed in companies of 20 employees or more, the allotment of budget to a National Fund for Disabilities, and the creation of a National Disability Advisory Council. The new Disability Act of 2010 holds great promise for improving the inclusion and quality of life of people with disabilities in Tanzania, if it is implemented as promised.

#### Contribution to the field

When attempting to understand the construct of intellectual disability in different contexts, speaking to family members in addition to the individual with the disability may provide new insight about understandings of and responses to intellectual disability in society and may help to identify the forms of support that are available or needed to ensure the quality of life of people with intellectual disabilities. This article reflects the shift from a deficit-based understanding of disability to a more positive, solutions-focused and strength-based understanding of disability.

### Literature review

The specific literature on family and intellectual disability in Tanzania is nearly non-existent, and is sparse on the continent in general (Njenga 2009). According to Kisanji (1995), who studied Tanzanian proverbs and what they reveal about society’s understanding of disability, although proverbs occasionally demonstrate negative attitudes towards people with disabilities, in general they demonstrate that society is tolerant, respectful, and in favour of care and assistance for such individuals. A recent study examining family perceptions of caring for children and adolescents with intellectual disabilities identified that areas of support required for families in Dar es Salaam were not only basic needs (e.g. daily activities and caregiving in the home such as feeding and toileting), but also education, healthcare, security, and economics (Mbwilo, Smide & Aarts 2010). This study by Mbwilo *et al*. concludes by identifying issues that could primarily be defined as deficits: deficits in family knowledge, deficits in economic capacity, and deficits of community or home-based health care programmes. Although identifying challenges, needs, and areas for improvement is important, researchers should also attempt to identify family strengths when conducting research on family responses to intellectual disability. As Summers, Behr, and Turnbull (1989) argue, families who successfully meet the challenge of a child with a disability have much to teach others about what works and about society’s own attitudes toward people with disabilities; and by focusing on a family’s distress, we provide less opportunity to build on family strengths as an intervention strategy.

## Methods

### Material and setting

Although my sample size of interview respondents was 13, it is also important to note that, similar to many activities occurring in a family home, during the interviews there were many other individuals (e.g., individuals with disabilities, aunts, adult siblings, grandparents, spouses, neighbours, and cousins) coming and going from the room, listening to the conversation, and occasionally corroborating the responses of the primary respondent (e.g. after a mother states that her daughter would like to be married one day, the daughter agrees that she would indeed like to be married). Usually, two or three times in an interview session, these extra family members would also provide their own perspectives on questions after a respondent had the chance to answer (e.g. when a mother responds to a question about the individual with disabilities’ strengths by saying that she loves how well her daughter looks after the neighbours’ children, a grandfather adds that his granddaughter is also very neat and clean). These insights from surrounding family members enriched the data I gathered from my primary respondents and gave me greater insight into family and household dynamics.

### Data collection

With the exception of four family units (who preferred that the interviews be conducted elsewhere), I held all interviews in the respondents’ homes. The households represented a wide range of different family structures: single mothers, two-parent households, grandparent-headed households, and households comprising a number of family members (e.g. sisters, brothers, cousins, aunts, uncles) under one roof. I conducted interviews either in English or in Kiswahili with the assistance of a translator. I chose the language in which I conducted the interview based upon interviewee’s preferences; approximately half of the participants chose to respond in part or entirely in Kiswahili, but the majority of interviewees seemed to understand questions when posed in English and responded in Kiswahili even before the question was translated. It is important to note that, due to my initial sampling procedures, which relied on the membership base of TAMH, my sample is likely skewed toward (a) persons who are inclined toward self-advocacy and advocacy of the rights of persons with disabilities (in terms of education about rights, empowerment and/or the time and resources available for advocacy efforts and membership in TAMH) and in some cases, (b) persons who are educated enough to converse in English (at least to secondary level but often to university level).

Interviews lasted between 30 minutes and an hour and 30 minutes. Interview questions focussed upon general family characteristics and goals for the future; how the family came to understand that their member had an intellectual disability, and what, if any, difficulties they have faced, lessons they have learned, or needs they have that are unmet. These questions are presented in the full interview protocol (Box 1). Initially, although the interview protocol included specific questions addressing strengths (e.g. ‘What do you think your family does particularly well?’ ‘What could I learn from your family that I could use to help other families in my country?’), families did not explicitly identify their strengths, thus I modified the protocol to reflect what is described above, and decided that I would have to utilise a grounded-theory approach in a discussion of my findings to identify family strengths from general, life-history-type stories.

BOX 1 Interview protocol questions.

**Table:** 

Please tell me about yourself and your family?
How did you come to realise that [family member] had a disability?
Why did you decide to join TAMH?
In general, what do you see as the role of parents in the life of a child with a disability?
What do you like most about [family member]?
What does your family do particularly well? What are your strengths?
What has been the greatest support for your family?
If someone had a family member with a similar disability as [family member], what would you like for them to know?
Have you ever been involved in disability advocacy efforts? [If yes] Could you please tell me about times when your efforts have been particularly successful?
Could you please tell me about times when your efforts have been particularly unsuccessful?
What are your dreams [for the family; for the individual] in the future?
I am interested in learning lessons from Tanzanian families of children with disabilities to help families in my home country. What could I learn from your family?
Is there anything that I have not yet asked you but that you think would be important for me to know?

I took detailed notes on a pre-established interview sheet and set aside time after each interview to type up these notes and fill in any further comments or observations about the interview into an electronic file. In my hand-written interview notes, when I took down a direct quote, I noted this with quotation marks. All other notes without direct quotes were close approximations of participant responses. In addition to participant responses to interview questions, I also kept field notes in which I entered detailed descriptions of the interview setting (typically of the home) and of the various family members present during my home visits and any notable family interactions (e.g. a grandfather affectionately pats his granddaughter’s leg as he proudly interjects his opinions about her strengths and accomplishments).

### Data analysis

My strategy for data analysis was consistent with Warren and Karner’s (2010:216) assertion that data analysis should begin during the data collection process: ‘[a] reading or relooking process should occur throughout the data collection process; preliminary analysis and analytic ideas should be noted at all phases’. I conducted the analysis concurrently and recursively within and across observations and interviews, using the constant comparative method (Charmaz 2006; Patton 2002) to identify themes as they emerged. Throughout the data collection process, I engaged in reflection and preliminary analysis of data as I entered data into computer files. I entered any preliminary observations and analytical memos about both interviews and field notes as ‘comments’ inserted into a word document. I triangulated interview responses with observations and field notes and consciously searched for negative cases, or contradictory observations.

Upon leaving the field, I read and reread interview and observation notes twice before identifying tentative themes. I then grouped responses together under tentative themes for further analysis and identification of sub-themes. I was primarily interested in family interview question responses; however, I used my observations to supplement the data provided in interview responses. Once themes and sub-themes were near finalised, I then coded my interviews and observations using a scheme of numbers and letters to designate the major categories and sub-categories in the data. I coded hard copies of all computer files of data using coloured pens to mark the margins with the appropriate numbers and letters. In between the coding of data and the writing of this paper, I made analytical memos which greatly assisted me in preparing to write a first draft, increasing the fluidity and depth of my writing, and enabling me to see how categories are connected in a larger, overall process (Charmaz & Mitchell 2001).

## Results

**FIGURE 1:**
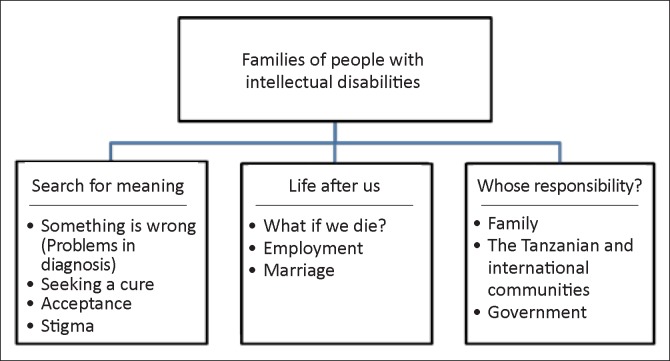
Results: Three themes of Dar es Salaam family experiences with intellectual disabilities.

Having outlined my methods for data collection and analysis and potential limitations and areas of researcher bias, I will now turn to a discussion of research findings. These findings will be organised to align with the major themes and sub-categories identified: (a) ‘search for meaning’; (b) ‘life after us’; and (c) ‘whose responsibility?’. See figure 1 for a visual representation of key themes. Following the presentation of findings, I will discuss the family strengths that can be drawn from these various themes.

### The search for meaning

In the early stages of finding that a family member has an intellectual disability, a number of themes arose amongst this study’s participants. With many of the families interviewed, during the early stages of understanding their family member’s disability, there was first a health crisis (e.g., seizures), or the notion that something was ‘different’ with the child. This was generally followed by a search to understand exactly what the health complication or ‘difference’ was. Next, some families discussed their own process of searching for a way to reverse or minimise this difference. Often, following this search, families were able to accept the person as they are. At the back of this search for meaning, there is often the sense of stigma surrounding the exceptional family member’s difference.

#### Something is wrong… (problems in diagnosis)

The families identified two different ways in which they came to understand that their child had an intellectual disability. First, a number of families told me that their child had had a normal birth and that they attributed their child’s disability to being an after-effect of some sort of health condition, such as seizures or suffering from cerebral malaria at an early age. Some families did not attribute their member’s disability to a particular illness. As one father stated, he went from hospital to hospital but nobody could tell him why his son was nonverbal and exhibiting challenging behaviours at the age of three. It was only after being seen by several doctors that the son was eventually diagnosed with autism.

#### Seeking a cure

Respondents often shared that in the early years, after learning about their child’s exceptionality, they searched for a ‘cure’. Whereas one father told me that if parents are educated they will look for scientific and Western cures and if they are not educated they will turn to traditional beliefs, witchcraft, and religion to search for a cure, this statement was contradicted by an interaction that I had with a mother who holds a Master’s degree. She told me, ‘many parents opt for traditional healers, because it [finding out one has a child with an intellectual disability] is a spiritual and superstitious thing’. When I asked her if she personally went to a spiritual healer with her son, she replied: ‘Of course! I am African! Everything, you try! You try everything!’ In this sense, I found Tanzanian discussions of a search for a cure not dissimilar from practices of medical pluralism found in Western families who seek out a second opinion from a different doctor or employ nutrition advice (e.g., gluten-free diet as a cure for autism).

#### Acceptance

All of the family members seemed to accept that their child had an intellectual disability and had stopped aggressively seeking a ‘cure’ (while still supporting their development through education, necessary healthcare, etc.). As one father stated, ‘It was a shock to my family, before. You are expecting that he will go to university and so you will worry that he will fail in life. But after time, you realise this is not such a big deal. It is normal.’

The theme of acceptance was also apparent when I asked respondents what they would tell parents who had just found out that their child had a disability similar to that of their own family member’s disability. For example, one aunt responded that she would tell the family that ‘it [the disability] is God’s plan, God’s wish,’ thus indicating that it was important to accept the child as they are. A mother in a different family responded, ‘First of all, it is acceptance. Acceptance comes with knowledge.’ She went on to express that many of the people who are around at the birth (e.g., doctors, midwives) are not trained in how to counsel families when a child is born with a disability. She suggested that adequate counselling would provide parents with knowledge about the disability, which would then assist in fostering their acceptance of their child and their disability.

#### Stigma

In addition to the sub-themes of ‘something is wrong’, ‘seeking a cure’, and ‘acceptance’, respondents discussed understanding their family member’s disability in a setting characterised by ‘stigma’. Families discussed stigma in a number of ways. First, respondents asserted that Tanzanians have a culture that looks negatively on people with disabilities and that characterises people with disabilities as a ‘bad omen’ for the family. On the individual level, parents discussed watching their children interact with others in the community. One father told me ‘people with mental disabilities live a subhuman life. They are not seen in the community’. Another father described on a number of occasions that children with intellectual disabilities are targeted and killed by those practicing witchcraft in the community and thus he does not let his son wander on the streets on his own.

Other parents identified the stigma that was shown through the language used by people in the community, such as the use of the derogatory term *talia* for a person with an intellectual disability. Whereas some families showed anger at community members who called their children names, other families reported that the community has also been a source of support for their members with disabilities: ‘Many people in the neighbourhood accept him. They are very supportive. They have called him names and such, but they have been very supportive to him. They talk with him, and they assist him in crossing the street.’ Another mother told me, ‘some people are accepting of Aziza, and others are not. Those who are accepting do not give us anything, but they talk to her; they have relationships with her. Those who are not accepting, they do not beat her, but they speak bad words to her.’

Stigma was also shown through discussions of abandonment, both of parents who abandoned children because of their disabilities and of fathers who abandoned their wife and family because of the birth of a child with a disability. A large number of families shared anecdotes about other mothers with children with disabilities that they knew who had been abandoned by their husbands because of the child with a disability. Although my sample provides an overwhelming contradiction to stories of family (especially father) abandonment due to the stigma of having a child with a disability in Dar es Salaam, my respondents insisted that this practice was commonplace. In my sample, I only had two families who directly demonstrated instances of abandonment due to disability. In the first instance, the child with an intellectual disability lived with his grandparents who were the primary caregivers because, they said, the mother ‘dumped’ the child with them because of his disability. The father of the child (the grandparents’ son) comes to visit his son nearly every day. Another respondent, a single mother, recounted her husband’s abandonment:

‘I was once married. […] I had two daughters when he left us. Mary was two years old when she got cerebral malaria. When she got this, my husband got superstitious, and he abandoned me. Due to the stigma of disability, my husband abandoned me completely. I have support only from my parents to take care of my children. I have small income-generating activities (selling clothes, ice-cream, and juice at the marketplace) but even this does not support my family.’

Although families told me anecdotes of women left alone and destitute when their husbands abandoned them because of the stigma of having a child with a disability, wealthier families told me of stigma experienced in a different way. Some of the wealthier families mentioned that they were accused of witchcraft because of their child’s intellectual disability. Specifically, they are accused of sacrificing their child’s intellectual capacity or creating a child with an intellectual disability to gain riches in life. One mother identified a term, *dondocha*, which she said meant that a child started like any typical child, and then the parents take away the ‘normal’ traits of their child and use the child’s spirit to get rich. ‘Often, even if I work hard and earn and have things to show for it, people say “it is because she has that child” and not because I have worked for it.’

#### Life after us

When I asked families about their dreams for the future, many family members expressed hope about a high quality life for their member with a disability. Some families also expressed unease about the future, especially if they are no longer around to provide care and support for the individual with the disability. In this section I will discuss dreams and worries for the future, including dreams for employment and marriage of the member with a disability.

##### What if we die?

Many primary caregivers voiced worry about what life will look like for their member with a disability in their absence. As one mother shared, ‘For parents of children with intellectual disabilities, you don’t know the future. My son is 32. He is living with me, and he is not married like his sister. We don’t know how his life will be. We don’t know.’ A small portion of respondents also mentioned worries that extended family and relatives may take advantage of their children with disabilities, especially if parents were planning to leave their children with an inheritance (e.g., putting the house in their name):

‘My son needs food, clothing, shelter. I am running a small restaurant, but what if we die? His future is uncertain without us parents. We don’t know if his relatives will support him in the way that we are doing. He would have the house, but who knows what people would do to him. They might kill him.’

Interestingly, none of the persons I interviewed expressed the possibility of siblings as potential future caregivers following the death of the parents; however, had I probed this possibility more explicitly, perhaps parents would have also voiced the option of having siblings take over the caregiving responsibilities.

#### Employment

One way in which parents saw a bright future for their children with disabilities was through the possibility of their child and family member holding gainful employment. A number of families had members with intellectual disabilities in young adulthood. All of these families disclosed that following completion of school, their member with an intellectual disability now just sits at the family home and does nothing. A number of these families noted that their members’ attitudes and abilities had been negatively affected after having been out of school for a number of months. Families argued that in an ideal world, after school, their family members should get employment; however, this was never the reality for the adult individuals with disabilities I met during this study.

As the following narratives indicate, when asked about their dreams for their future, a number of different families expressed the goal of employment:

‘My son is nonverbal, so I worry about this. If he could be independent, if he could wake up and go do some work, that would be enough for me.’

* * *

‘I see her able to work in a domestic environment, cleaning, cooking, etc. She helps me wash clothes and iron. She also looks after the other children.’ [Note, the daughter of this mother told me that her employment dream was to be a fashion model.]

* * *

‘My son, he can do physical work. He is very strong so as long as it is not dangerous (nothing sharp, no electrical wires) like stacking boxes or something, he could work, and the employer might not even know that he has a disability.’

Finally, similar to the quotes above, as one father said of his son: ‘We would like for him to be a professional, have his own life, his own family.’ In addition to mentioning the importance of becoming employed, respondents also expressed that marriage of the family member with a disability was a dream for the future. A number of families mentioned marriage both to ensure that family member’s fulfilment of what is considered a ‘normal’ life in society, and, in some instances, to ensure that the member with a disability will have others to care for him or her.

#### Marriage

At the conclusion of one interview, a family solicited my advice: What did I think about their daughter Aziza marrying? They voiced concern that, although they would like Aziza to get married, they worry that her husband may mistreat her due to her disability. They worry that they may not be able to protect her from a bad husband. A small number of families of persons with disabilities expressed a similar concern. However, based upon what the family members said in the interview, I had come to learn that, although there was worry about Aziza’s wellbeing, marriage was an important dream, both for the family members and for Aziza. Aziza’s mother and her aunt shared: ‘Our dream is that she should be married. So that when we die (the mother and the aunt), Aziza will not be alone. She can have children to help her.’ When I followed up with Aziza and asked her what she thought about marriage, she (in contrast to her shy and evasive manner toward me in general) happily and enthusiastically told me that she would, one day, like to be married.

Similarly, one father of a son with disabilities expressed his desire for his son to marry so that he might live a normal and dignified life, typical of any other person in Tanzania. ‘I would like for him to get married. He is fond of girls and he understands what marriage is. I would like for him to find a wife so that he can live with respect and dignity.’ Marriage seemed like a desired life goal for most families with members at or reaching adulthood.

#### Whose responsibility?

A final theme that ran through the interviews was that of responsibility. Whose responsibility was it to provide support, both to the individual with the disability in specific and to the family in general? In general, respondents seemed to attribute the responsibility for the wellbeing of individuals with disabilities to the entire society as a whole. However, often responses indicated that respondents attributed responsibility to various segments of society, specifically to the family unit, the Tanzanian community, the government, and the international community.

#### Family

It was a general sentiment that family is the most important support in the life of a person with a disability; thus, the family had an important responsibility to provide for their members with disabilities. As one parent noted: ‘Parents are very involved in the lives of people with intellectual disability because parents are all these children have. If you are lucky, you also have siblings.’ In terms of assisting primary caregivers to support the individual with the disability, primary caregivers (usually mothers) often identified other family members as important sources of support in their lives. Mothers who were still with their husbands (e.g. they were not divorced or widowed) often identified them as huge sources of support in fostering family quality of life. Others identified other family members as important supports: ‘My young sister has stayed with my son when I travelled. She has been a big support. My son likes her a lot. He even calls her mama. Even the maids have been a big support for me.’

Two mothers told me that other families with children with disabilities have come to rely on them, as seasoned parents seen as having valuable experience, for sources of information and support:

‘Because people know me, now they are coming to my house to ask for my advice and help with their children’

* * *

‘Parents who come for advice are few. Many just give up. But those who do come are interested in free medication. Education. But most parents just give up. There was a neighbour who was hiding his child in the house. He saw Mary and came and asked if she went to school and where. Then he started to send his child to school.’

The data indicated that families had a number of responsibilities in terms of support for people with intellectual disabilities. First, parents had a responsibility to care for their child with disabilities. Next, the extended family often provided support but with less of the ‘responsibility’ to provide support. Finally, seasoned family members were also sometimes responsible for supporting other parents in fulfilling their role in providing for their children with disabilities. In addition to family responsibility to support the quality of life of people with disabilities, some families believed that the wider Tanzanian society and even the international community had obligations to provide support.

#### The Tanzanian and international community

In conversations, families often brought up the various Tanzanian organisations which they expected to provide services for them. For example, families identified churches and religious organisations as responsible institutions for welcoming and supporting individuals with disabilities. Families also identified national non-governmental organisations (NGOs) and civil society organisations (CSOs) as key providers of economic and social support. One father lamented that, although everyone in Tanzanian society has an obligation to support its most vulnerable members, most were not fulfilling this obligation:

‘The world over, civilisation is taking care of disabled people. If people with disabilities are eating from trash like dogs and cats, that’s not civilisation. We must organise our support for people with disabilities in Tanzania.… We can learn from other countries and other examples, so that maybe we won’t make the same mistakes that they have.’

The last sentence of this father’s quote hints at the next sub-theme of responsibility: the international community. A number of families identified various forms of support provided by the international community that were important in their lives. These included the free healthcare provided in the Comprehensive Community Based Rehabilitation Tanzania (CCBRT) disability hospital, and various income-generation and microfinance support programmes from international NGOs. Families spoke about these supports as if the international community had a responsibility to provide them and the families had a right to access them. For example, when talking about her son’s inclusion, one parent stated: ‘Government, NGOs, CBOs, should make sure that people with disabilities have the opportunity to participate in all aspects of life-daily activities and economics as well. They [people with disabilities] can do it; they just need the opportunity for participation.’

Finally, as the above quote indicates, a number of families asserted that the government had a great responsibility for providing for people with disabilities and their families.

#### Government

In spite of various initiatives that the Tanzanian government has implemented in support of people with disabilities, many family respondents felt abandoned by a government that had not fulfilled its responsibility. As one parent said:

‘There is a lack of government support. [TAMH exists] because outside organisations are assisting us, but there is no government allotment of funds. We depend on begging for donors. For me, that is key because you can’t have an organisation without [*funding*].’

Another parent argued that the government has an unfulfilled responsibility to provide disability prevention and parent education once an intellectual disability is identified. A great number of arguments were levelled that insisted the government was not fulfilling its civic responsibility:

‘When you listen to some government officials, they speak as if it is the responsibility of NGOs and civil society to care for these groups, when this is not the case. It is too big for them. We also need government support.’ (Father of child with ID)

* * *

‘Life in general, here in Africa, especially for families … families of people with disabilities in your country, in Canada, in America, they typically get support from the government. But here we get no support. Here in Tanzania, it is your own problem.’ (Mother of adult with ID)

Other families felt deceived that the government had promised them various forms of support but that they had so far been unable to access them:

‘My son, he takes medication morning and night for epilepsy. I spend 35 to 40 US dollars a month on his medication. I pay for it all. I have no support from the government. It says in the government policy that they should support us, but up to now, I have not seen any of this support. It says in the policy that they are supposed to do this, but I have not seen them do this.’

* * *

‘The local government, they said they would help, and that was six years ago and they have still not helped us. There has been no support from the government and we go and return and go and return to the municipal government office without any result.’

Although a number of parents reported that the government does not provide promised supports (e.g., free health care and/or medication for individuals with disabilities), conversations with other families and healthcare providers indicated that the government did indeed provide its promised support. As one mother told me, ‘At Temeke hospital, they are giving me service freely. The government pays for it.’ As this discrepancy demonstrates, the problem may not be that the government is not providing its promised support; it may be that families just did not know how to access this support.

As previously mentioned, because respondents were a part of a self-advocacy organisation, many of them are inclined toward social movements and social action. It is likely that the above-described sense of being let down by the state plays a role in engendering social action. For example, the Tanzanian Association for the Mentally Handicapped (TAMH) is part of a wider umbrella organisation of various DPOs that engages in significant national lobbying and advocacy efforts for the rights of persons with disabilities. Moreover, through their active involvement in the TAMH, a number of parents interviewed had been directly involved in the consultation process for the creation of the new 2010 Disabilities Act.

## Ethical considerations

Before entering the field, I obtained ethics approval for this project from the University of Kansas’ Human Subjects Committee. One aspect of this approval was the guaranteed confidentiality of my informants, thus every name in this paper has been changed to reflect this commitment to confidentiality. Once in the field, I recruited participants through the TAMH. Participant recruitment included anyone who self-identified as a family member of a person with an intellectual disability. Because I relied on self-identification of participants, some participants had children or relatives with a formal diagnosis of an intellectual disability, whereas others had family members who did not hold a formal medical diagnosis of intellectual disability. TAMH leadership identified 13 of its members who were willing to take part in home visits and interviews. This identification and selection of members to interview was based upon members who were active in the organisation, willing to welcome a foreign interviewer, and who had ability to provide an interview on short notice. Additionally, I selected only those persons who self-identified as the primary caregiver or guardian of the individual with the disability to be the primary interview respondent.

For all home visits, a representative of TAMH accompanied me and facilitated introductions with the families. This study comprises data obtained from interviews with a total of 12 different family units with 12 individuals with disabilities (8 males, 4 females; ages 4–32). The interviews were arranged with individual caregivers or guardians of the person with the disability; these caregivers were the primary target of and responded to all interview questions. Because in one family, a primary-caregiving couple (two individuals) participated in answering all questions together, this study’s sample size is 13 individuals from 12 households: 3 fathers, 8 mothers, and 1 grandparent couple.

## Discussion

Having outlined the key findings of the interview, I will now attempt to understand these responses by applying theories of family strengths to the various themes identified. Finally, I will conclude by suggesting ways in which to expand upon these findings in future studies. DeFrain (1999) classifies family strengths in the following categories: commitment; appreciation and affection; positive communication; time together; spiritual wellbeing; and the ability to cope with stress and crisis. The data demonstrate a variety of such strengths, upon which I will expand. Although many respondents demonstrated all of these strengths in subtle ways (i.e. enjoying spending a great deal of time together and verbally and non-verbally expressing appreciation and affection), this discussion will focus on three of the most salient strengths present in interview responses. These strengths include coping, spiritual wellbeing, and commitment.

### Coping, spiritual wellbeing, and commitment

A large number of families discussed their thought process in the early years of attempting to understand and negotiate intellectual disability. This process often fell into sub-categories of learning that ‘something is wrong’, seeking a cure for disability, and finally, acceptance. Theory suggests that seeking out a causal attribution for situations of stress or for aversive experiences may help individuals to gain or re-establish a sense of control (Taylor, Lichtman & Wood 1984) or a sense of orderliness or predictability within one’s environment (Rothbaum, Weisz & Snyder 1982). When participants described instances of searching for understanding what was ‘wrong’ for their child and seeking a ‘cure’ from numerous sources, family members were displaying behaviour that is aligned with positive coping and adaptation skills (Summers, Behr & Turnbull 1989). Furthermore, family ability to cope with stress and crisis are shown when families eventually re-establish balance and accept their family member as he or she is, while still promoting that member’s positive development, as shown in the sub-theme of ‘acceptance’.

DeFrain (1999:11) describes the elusive concept of ‘spiritual wellbeing’ as ‘connection to each other and connection to that which is sacred to us in life’. Spiritual wellbeing can be manifested in religious terms, harmony, or ethics. Perhaps most notably, the strength of spiritual wellbeing was shown when families discussed acceptance of their member with a disability. Acceptance was sometimes spoken of in religious terms (e.g., ‘It is God’s will’) but was also spoken of in terms of love and connection to the family member with a disability. One may also identify spiritual wellbeing in those whose responses attributed responsibility of supporting an individual to the society as a whole. This sort of attribution of general societal responsibility to care for those in greatest need demonstrates a strong sense of ethics and of the interconnectedness of the community.

The strength of commitment is demonstrated in a variety of ways in interview responses. When families are committed, they do not let outside influences (e.g., work, or other priorities) take away from family interactions. Families with the strength of commitment view their life together as a family to be of utmost importance. Commitment was most salient in interview responses in the ‘whose responsibility?’ theme. First, respondents viewed themselves as responsible for keeping the family together and for caring for their family member. Fathers with whom I spoke provided examples of fathers who certainly had not abandoned their family as a result of having a child with a disability, even though a number of respondents reported that father abandonment is common for children with disabilities in Tanzania. Next, respondents’ identification of and advocacy for support needs hinted at commitment in a more subtle (but not less important) way. All of the families with whom I spoke were so committed to their family and their member with an intellectual disability that they went out of their way to become advocates for their family’s needs (e.g., first evidenced through their involvement in the parent advocacy organisation, TAMH). Some of the families had shown their commitment by being involved in and having a voice at national disability policy drafting efforts; one mother demonstrated her commitment by continuously going to the municipal government office in an attempt to gain support for her family member with a disability. Thus, I conjecture that the family strength of commitment is not only manifested in vocal assertions of the importance of family but also in the specific actions of self-advocacy that members undertake in order to meet the unique needs of their family.

In addition to understanding family strengths, it is also important to note that the issues of stigma, feelings of hopelessness, and lack of formal support that were presented in these interviews could be linked to the literature on caregiving burnout or stress. As mentioned in the introduction to this article, research on parental stress has noted that providing care for a family member with an intellectual disability often requires additional physical, emotional, social and financial resources and that it is necessary to coordinate the family member’s unique needs while balancing competing family needs (Murphy, Caplin & Young 2006; Silver, Westbrook & Stein 1998). Caregiving, then, has been seen to contribute to parental stress and to lower caregivers’ sense of psychological wellbeing (Cramm & Nieboer 2011). But, knowledge of the ways in which families experience stress (in this case through stigma, feelings of hopelessness, and lack of support) can inform the utilisation of family strengths to create appropriate support. For example, it has been found that formal and informal support can act as a buffer, with parents reporting lower stress, anxiety and depression when they perceived greater support (Blacher, Neece & Paczkowski 2005). Having discussed some of the potential strengths that may be identified from family discussions of their experiences with intellectual disability, and additional stressors, I will now identify some potential implications of these strengths on future interventions and identify areas for future research.

#### Implications for future interventions

Based upon the above discussion of family strengths, two immediate implications for future practice are apparent. Firstly, healthcare providers and other practitioners should support and encourage families as they search for cause of their child’s exceptionality. Rather than withhold all but ‘necessary’ knowledge about the nature of the child’s impairment or discourage ‘shopping around’ for solutions, practitioners should support families in their efforts to understand the nature of their child’s impairment. Supporting this search for meaning may build upon and advance family strengths in coping with stress and crisis.

Next, practitioners might try to build upon the strength of commitment shown by many of the strong family advocates in Dar es Salaam, such as those who participated in this study. Family advocates have the potential to serve as important resources for families of children with similar disabilities, such as the two mothers in this study who described already doing this on an informal basis by providing advice on such issues as education and healthcare to other families with children with disabilities in the neighbourhood. In capitalising upon the strength of commitment, seasoned family advocates may be able to provide information to new families to help them identify and access available resources. For example, although some parents were accessing free government healthcare and medication, others had heard of this support but had been unable to figure out how to access it. A more formalised system that facilitated the sharing of expertise of committed family advocates may help to alleviate problems due to lack of information. Research has shown that families in Africa have an important role to play through advocacy, education on human rights, empowerment, and development of policies about intellectual disability (Ngatunga 2004), and I suggest that families who demonstrate this strength of commitment will be able to lead the way.

## Conclusions, limitations, and suggestions for future research

In this study, I have attempted to probe family understandings of and experiences with intellectual disability in Dar es Salaam. The most salient themes that arose from this research included searching for meaning, life in the future, and responsibility to provide for people with disabilities. Although it is important to identify family concerns, needs, and areas for improvement, it is also important to identify and value family strengths in order to respectfully support families and to identify effective solutions to problems. Building upon the qualitative study of family experiences and support needs conducted by Mbwilo *et al*. (2010), this study probed family responses to disability and family needs and applied DeFrain’s (1999) classification of family strengths to interview responses.

Across interview responses, I was unable to identify any systematic differences in responses based upon gender (of respondent or individual with a disability) or on socioeconomic status of the family; however, it is possible that these differences exist and my sample size was simply too small to enable this identification of systematic differences. Future study may seek out specific examples of differences in gender or socioeconomic status and the implications this has for the disability experience or disability advocacy efforts in Tanzania. Future research may also examine the developmental considerations of a family’s experience: Does the age of the child impact the family experience? What are the changing health, educational, and social needs of a family as a child develops? What is the role of families in each life stage or during transitions between life stages?

Additionally, future research that attempts to further probe specific family strengths and create interventions based upon these strengths will be important. For example, in this study, most of the families experienced stigma in various forms. It would be useful to adopt a family strengths approach to understand how parents are presently utilising their strengths to navigate this stigma or to initiate supports which aid families to draw from their unique strengths to navigate or even eliminate the stigma that they encounter in society.

Lastly, and perhaps most importantly, the voice of people with intellectual disabilities themselves is too often absent from literature on intellectual disability. One area of utmost importance in future research will be to increase involvement of people with intellectual disabilities themselves in intellectual disability research in Tanzania. This study, which focused upon *family* response to ID as opposed to *individual* response to ID, cannot be exempt from critiques of representation and voice, and I acknowledge that future studies must strive to provide a voice to people with intellectual disabilities and to incorporate their viewpoints to describe how they understand their own disability and to identify and build upon their personal strengths.
